# Determinants of unmet needs for mental health services amongst adolescents in Shiraz, Iran: a cross-sectional study

**DOI:** 10.3389/fpubh.2024.1265611

**Published:** 2024-02-06

**Authors:** Hassan Joulaei, Maryam Fatemi, Razieh Joulaei, Mohammad Reza Heydari, Ahmad Reza Pourmontaseri, Zohre Foroozanfar

**Affiliations:** ^1^Health Policy Research Center, Institute of Health, Shiraz University of Medical Sciences, Shiraz, Iran; ^2^HIV/AIDS Research Center, Institute of Health, Shiraz University of Medical Sciences, Shiraz, Iran; ^3^Department of Psychology, Shiraz Branch, Islamic Azad University, Shiraz, Iran

**Keywords:** mental health services, unmet needs, schemas, adolescents, Iran

## Abstract

**Background:**

Mental disorders are increasingly prevalent among adolescents without appropriate response. There are a variety of reasons for unmet mental health needs, including attitudinal and structural barriers. Accordingly, we investigated perceived mental health needs, using mental health services, and their barriers in adolescents.

**Method:**

This cross-sectional study was conducted in 2022 in Shiraz, Iran. Demographic characteristics, the Adolescent Unmet Needs Checklist, and the Young Schema Questionnaire were administered to 348 adolescents aged 13–19 years. Adolescents were classified as having no needs, fully met needs, partially met needs, or wholly unmet needs. Logistic regression analysis was used to determine factors associated with perceived unmet need and refer participants to healthcare centers.

**Results:**

193 (55.5%) adolescents reported perceived need for mental healthcare out of whom, 21.6% reported fully and 21.6% partially unmet needs. Noticeably, only 12.4% of needy participants reported met need. “Reluctance to seek mental healthcare” and “asked but not receiving help” were common barriers to using the services.

**Conclusion:**

The present study reveals unmet mental healthcare needs as a significant public health concern among the adolescents. To address this significant concern, reorientation of primary care, removing economic barriers from mental healthcare services, and improving health literacy in the community are recommended.

## Introduction

Adolescence is a period of transition from childhood to adulthood, during which teenagers undergo psychological, physical, and social changes (1). According to the World Health Organization (WHO), adolescence is defined to range from the age of 10 to 19 years old (2). This definition applies to approximately 50% of mental health conditions that begin before the age of 14 (2) and 75% of mental health conditions that begin before the age of 18 (3). Obviously, meeting and identifying psychological needs of adolescents, according to psychological theory, results in the successful passage of their one or more developmental stages (4). On the other hand, failure to meet mental healthcare needs of adolescents will results in particular developmental problems such as mental disorders or EMS. However, some needs are not met completely while others are totally ignored (for example, respect for human rights in societies afflicted by racism). Over the recent years, social psychologists have coined a new term to describe unmet needs of the human psyche when debating social and environmental constraints (5). According a systematic review, in spite of mental disorders’ prevalence among adolescents, the utility and quality of mental health services is limited, especially in low resource settings. It also revealed that privacy, confidentiality and patient- centered care, and presence a responsive therapist to their needs were important factors for adolescents to utilize mental health services. They would rather avoid judgment and embarrassment and fear that their mental health status will unfold for others (6).

Unmet needs are defined as the gap between the services required to address one’s health concerns and the services one receives (7). While these needs exist and have been expressed at specific points in time, they have been overlooked by friends, family, and even policymakers for various reasons, including stigma, socioeconomic deprivation, and socio-emotional deprivation (3, 8). Among these needs, we could mention mental health services (MHS) and the need for counseling, which are far less likely to be met at low socioeconomic status (SES) than at other levels of society. Adolescents in need of additional psychological support often have a reduced likelihood of actively seeking it (9).

There are four determinants for using health services: acceptability, accessibility, affordability, and availability (10). Understanding the barriers and unmet needs of adolescents are critical for advancing adolescent support programs and timely interventions. Previous studies have indicated that in addition to various dimensions of accessibility associated with the health system, individual tendencies influenced by sociocultural factors and prior service experiences affect receiving services (11, 12).

Evidence has revealed a higher prevalence of unmet health service needs in Asian countries. Previous studies in Pakistan and Thailand revealed that factors like low-quality services, insufficient government support, and family culture were significant determinants associated with the accessibility to health needs (13, 14). Furthermore, a study conducted in India indicated the highest unmet health needs among women (15). Moreover, global studies have reported the highest rate of unmet needs in African countries (16). In Iran, previous studies indicated that residence location, employment status, access to media, level of education, and standard of living are associated with unmet health needs (17).

To the best of our knowledge, no study has been conducted on unmet mental health needs in adolescents in Iran. It is obvious that assessment of unmet needs is a straightforward method for determining the accessibility, utility and degree of inequality in providing care. Thus, this study was conducted to assess perceived unmet mental health needs in adolescents, MHS use, and structural and attitudinal barriers to using these services in Shiraz, as the fourth most populous city in Iran.

## Materials and methods

### Participants and procedures

This cross-sectional study was conducted in Shiraz, a city in Southwestern Iran with an adolescent population of 22% of the total population (1,800,000), in 2022. Based on the results of a pilot study in 50 samples, 348 adolescents aged 13–19 years voluntarily participated in this study. Female and male adolescents aged 13–19 living in Shiraz were included in the study. Adolescents with psychosis, cognitive disorders, or severe communication difficulties, which prevented their parents or legal guardians from signing an informed consent form were excluded from the study. For data collection, convenience sampling method was used. This sampling method have some advantages including collect data quickly, inexpensive to create samples, and readily available sample. However, this method can have disadvantages, the most important of which is bias in sampling.

To ensure a representative sample, we selected various public venues from all the ten zones within Shiraz Municipal Divisions. These venues included parks, coffee shops, shopping malls, and private English language sessions, providing services for a diverse socioeconomic spectrum where adolescents are frequently gathered. Two experienced psychologists served as interviewers (MF and RJ) approaching potential participants in these public venues. Initially, given the inclusion and exclusion criteria, the interviewers checked the willingness and ability of the adolescents to participate in the study and accurately respond to the questions. Then, they explained the objectives and significance of the study and requested the consent of the parents for participants’ under15 years old, or obtained the consent of participants with 15+ years of age. The consent form was carefully read and signed by the parents or the participants, as appropriate. Interviews conducted in a private place in the venue, away from the public and, most notably, their peers to guarantee the confidentiality of the information and encourage improved collaboration of the participants.

Demographic and behavioral data, as well as adolescents’ data on unmet mental health needs (perceived unmet needs, MHS use, and structural and attitudinal barriers for receiving needed services) were collected using a researcher-made checklist. To evaluate their early maladaptive schema, Young Schema Questionnaire (YSQ-S3) was used.

### Measures

#### Demographic and behavioral characteristics

According to literature review, variables related to unmet needs of mental health were extracted in other studies and the following variables were collected in this study.

The demographic variables included sex (male/female), age, level of education (middle school, high school, and college), parents’ level of education (Elementary/Diploma/Associate or Bachelor/Master or PhD), and parents’ job. Moreover, self-report Yes/No questions on behavioral variables included smoking, drug use, alcohol use, hookah use, and having a friend of the opposite sex, and socioeconomic status (SES) were asked.

#### Adolescents’ perceived unmet mental health needs

Perceived unmet needs refer to the needs recognized by an individual but have not been addressed due to multiple reasons. To assess perceived unmet mental health need, respondents were asked “Have you ever felt the need for mental health treatment or counseling but did not receive counseling for mental illness during the past 12 months?” (Yes/no response). Also, service use was assessed by the question “Have you had a consultation with any Counselor or psychiatrist for mental health problems during the past 12 months?” (Yes/No response).

**Figure 1:**
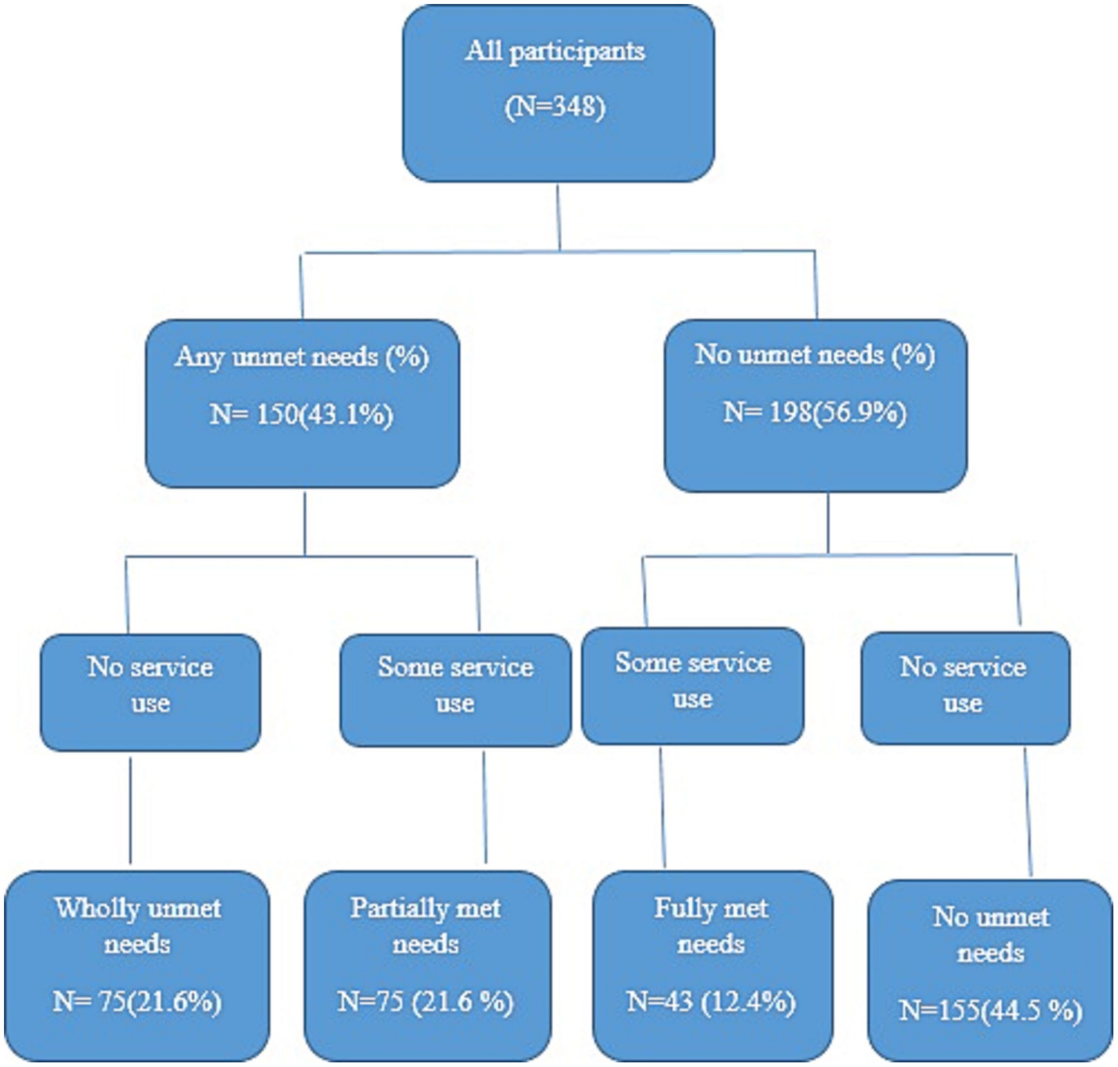
Unmet mental health needs classification diagram.

Based on the method of Meadows et al., adolescents who answered “Yes” to the first question were classified as having “any unmet need.” Adolescents with any unmet need who reported MHS use in the past 12 months were classified as having “partially met needs” and adolescents with any unmet needs who had no MHS use were classified as having “wholly unmet needs.” Also, adolescents who answered “No” to the first question were classified as “No unmet needs.” Adolescents with no unmet need who reported MHS use in the past 12 months were classified as having ‘Fully met needs’ and adolescents with no unmet need who had no MHS use were classified as having “no unmet needs” (Figure 1) (18).

#### Barriers to care

Structural and attitudinal barriers for receiving needed services were examined in participants who perceive “Any unmet need” with the question “Which of the nine barriers kept you from getting the help you felt was needed?”

##### Structural barriers include

Lack of financial means or being unable to afford the money, lack of insurance or insufficient coverage for services, transportation problems to receive a service, asking but not receiving help, and getting help from other sources.

##### Attitudinal barriers include

Presence of stigma (being afraid to ask for help), low perceived efficacy of treatments, not knowing where to get help, thinking that nothing could help, and reluctance to seek mental health care.

#### The place for receiving services

The participants who had referred to receive services were asked about their referral places (school consultant, hospital, private clinic, specialized government centers, public health centers, behavioral counseling center, telephone consultation, and traditional healers).

#### The young schema questionnaire (YSQ-S3)

The YSQ-S3 is a self-report questionnaire comprising 90 items, which assesses 18 EMS (five items each) (19). Each item is rated on a Likert scale ranging from 1 (completely false about me) to 6 (describes me perfectly). A high score on a subscale index indicates that the assessed EMS is more pronounced. In several studies, the YSQ-S3 demonstrated adequate validity and reliability for predicting psychopathology (20, 21). The Persian Version of the YSQ-S3 has favorable psychometric properties when administered to students (22). Cronbach’s alpha was applied to determine the validity of the YSQ-S3 scale for all the subscales ranging from 62 to 90.

### Statistical analyses

The mean ± standard deviation of the quantitative variables was reported. Also, categorical variables were expressed as numbers and percentages. In order to determine the factors associated with the perceived unmet need for mental health and referring to a psychologist, Logistic Regression Analysis was used. Also, t-test was used to compare the mean EMS in the sub- groups. To analyze the data, SPSS software (version 22) and Graph Pad Prism software (version 8) were employed. Additionally, a *p*-value of <0.05 was considered to be statistically significant.

### Ethical considerations

Prior to data collection, all the parents or legal guardians were asked to provide an informed consent to allow their children’s participation. Also, all the participants signed a written informed consent on their right to withdraw from the study as well as the confidentiality of their data. This research was conducted following the review and approval by the Ethics Committee of Shiraz University of Medical Sciences (IR.SUMS.REC.1399.1065).

## Results

The study enrolled 348 adolescents, out of whom 187 (53.7%) were male. The mean age of the participants was 16.93 ± 1.72 years. A total of 72 (20.7%) adolescents reported smoking, 77 (22.1%) drinking alcohol, and 21 (6.05%) reported using drugs. Table 1 represents, in details, the demographic and behavioral characteristics of all participants. Overall, 150 (43.1%) individuals reported perceived unmet need for mental health care. Also, 118 (33.9%) adolescents were referred to a psychologist and received some services. In total, 75 (21.6%) adolescents were classified as having wholly unmet needs, 75 (21.6%) partially met needs, 43 (12.4%) fully met needs, and 155 (44.5%) no unmet needs (Figure 1).

**Table 1:** Demographic and behavioral characteristics of the participants by perceived unmet need for mental health care.

Variables	Total (*n* = 348)	Perceived unmet need for care (*n* = 150)	Have not unmet need for care (*n* = 198)
Age	16.93 ± 1.72	17.05 ± 1.64	16.85 ± 1.78
**Sex**			
Male	187 (53.7)	87 (58.0)	100 (50.5)
Female	161 (46.3)	63 (42.0)	98 (49.5)
**Education**			
Middle school	69 (19.8)	31 (20.7)	38 (19.2)
High school	244 (70.1)	102 (68.0)	142 (71.2)
University student	35 (10.1)	1711.3	18 (9.1)
**Head of household**			
Father	278 (79.9)	109 (72.7)	169 (85.4)
Mother	34 (9.8)	18 (12.0)	16 (8.1)
Other	36 (10.4)	23 (15.3)	12 (6.1)
**Live with**			
Father and mother	282 (81.0)	112 (74.7)	170 (85.9)
Only father	21 (6.0)	9 (6.0)	12 (6.1)
Only mother	30 (8.6)	17 (11.3)	13 (6.6)
other	15 (4.3)	12 (8.0)	3 (1.5)
**Father’s education**			
Elementary	40 (11.5)	27 (18.0)	13 (6.6)
Diploma	54 (15.5)	27 (18.0)	27 (13.6)
Associate/bachelor	220 (63.2)	80 (53.3)	140 (70.7)
Masters/PhD	34 (9.8)	16 (10.7)	18 (9.1)
**Mother’s education**			
Elementary	49 (14.1)	32 (21.3)	17 (8.6)
Diploma	73 (21.0)	31 (20.7)	42 (21.2)
Associate/bachelor	197 (56.6)	73 (48.7)	124 (62.6)
Masters/PhD	29 (8.3)	14 (9.3)	15 (7.6)
**Father’s job**			
Unemployed	12 (3.4)	9 (6.0)	3 (1.5)
worker	46 (13.2)	21 (14.0)	25 (12.6)
Employed	137 (39.4)	40 (26.7)	97 (49.0)
Free job	126 (36.2)	68 (45.3)	58 (29.3)
Retired	27 (7.8)	12 (8.0)	15 (7.6)
**Mother’s job**			
housewife	242 (63.5)	107 (71.3)	135 (68.2)
working at home with income	49 (14.1)	17 (11.3)	32 (16.2)
Employed	57 (16.4)	26 (17.3)	31 (15.7)
**Socio-economic status**			
Lowest	10 (2.9)	5 (3.3)	10 (5.1)
Low	45 (12.9)	40 (26.7)	69 (34.8)
Middle	169 (48.6)	67 (44.7)	102 (51.5)
High	109 (31.3)	31 (20.7)	14 (7.1)
Highest	15 (4.3)	7 (4.7)	3 (1.5)
**Smoking**	72 (20.7)	43 (28.7)	29 (14.6)
**Hookah**	77 (22.1)	41 (27.3)	36 (18.2)
**Alcohol**	100 (28.7)	58 (38.7)	42 (21.2)
**Drug use**	21 (6.0)	14 (9.3)	8 (4.0)
**Having a friend of the opposite sex**	133 (38.2)	64 (42.7)	69 (34.8)

Data reported as *N* (%), mean ± SD.

Table 2 shows the mean score of Schemas in Young Schema Questionnaire by perceived unmet need for mental health care as well as referring to a psychologist. The results indicated that the mean score of all the Schemas in the YSQ-S3 was significantly higher in individuals who perceived an unmet need for mental health care as compared with adolescents who did not. Also, the mean score for some Schemas in the Young Schema Questionnaire (i.e., Mistrust/abuse, Defectiveness/shame, Failure, Dependence/incompetence, Vulnerability to harm, Enmeshment/undeveloped self, and subjugation) was significantly higher in individuals who referred to a psychologist as compared with that in the adolescents who did not refer to a psychologist (Table 2). As compared with adolescents with partially met needs, adolescents with wholly unmet needs had higher scores of all the Schemas in the YSQ-S3.

**Table 2:** Mean of schemas in young schema questionnaire by perceived unmet need for mental health care and referred to a psychologist in all participants.

	Perceived unmet need for care (*n* = 348)		*p*-value	Referred to a psychologist (*n* = 348)		*p*-value
	Yes (*n* = 150)	No (*n* = 198)		Yes (*n* = 118)	No (*n* = 229)	
Domain						
Emotional deprivation	12.90 ± 6.01	12.05 ± 3.23	0.001^*^	11.28 ± 5.14	10.79 ± 4.81	0.382
Abandonment/instability	13.50 ± 6.22	12.07 ± 5.55	0.022^*^	12.69 ± 5.36	12.67 ± 6.15	0.973
Mistrust/abuse	14.16 ± 5.98	10.77 ± 3.77	0.001^*^	13.36 ± 5.69	11.64 ± 4.63	0.003^*^
Social isolation/alienation	13.10 ± 5.96	9.89 ± 3.42	0.001^*^	11.64 ± 4.96	11.07 ± 4.93	0.322
Defectiveness/shame	11.21 ± 6.15	9.15 ± 3.84	0.001^*^	11.08 ± 5.588	9.52 ± 4.71	0.007^*^
Failure	11.30 ± 5.89	9.58 ± 4.11	0.001^*^	11.32 ± 5.44	9.82 ± 4.73	0.009^*^
Dependence/incompetence	10.87 ± 5.36	9.49 ± 4.11	0.008^*^	11.21 ± 5.17	9.51 ± 4.47	0.002^*^
Vulnerability to harm	11.57 ± 5.81	8.80 ± 3.08	0.001^*^	11.21 ± 5.54	9.36 ± 4.02	0.001^*^
Enmeshment/undeveloped self	12.36 ± 5.87	10.50 ± 4.06	0.001^*^	12.11 ± 5.31	10.89 ± 4.77	0.032^*^
Subjugation	12.51 ± 5.58	10.36 ± 3.78	0.001^*^	12.66 ± 5.41	10.58 ± 4.22	0.001^*^
Self-sacrifice	16.37 ± 6.13	12.58 ± 5.33	0.001^*^	14.26 ± 5.92	14.18 ± 6.02	0.906
Approval/recognition seeking	15.45 ± 6.63	11.35 ± 3.73	0.001^*^	13.67 ± 6.55	12.81 ± 4.88	0.211
Emotional inhibition	14.47 ± 6.58	10.77 ± 4.38	0.001^*^	12.56 ± 5.77	12.26 ± 5.72	0.650
Negativity/pessimism	12.89 ± 6.01	12.06 ± 3.24	0.001^*^	11.27 ± 5.14	10.89 ± 4.81	0.384
Punitiveness	13.11 ± 5.97	9.88 ± 3.41	0.001^*^	11.62 ± 4.96	11.07 ± 4.93	0.321
Unrelenting standards	17.09 ± 6.16	13.96 ± 5.35	0.001^*^	15.42 ± 6.03	15.24 ± 5.86	0.794
Entitlement/grandiosity	17.04 ± 6.30	12.52 ± 5.11	0.001^*^	14.46 ± 5.76	14.46 ± 6.23	1.000
Insufficient self-control and/or self-discipline	15.41 ± 6.63	11.36 ± 3.71	0.001^*^	13.64 ± 6.65	12.85 ± 4.88	0.215

Data reported as mean ± SD.

Table 3 summarizes the factors associated with perceived unmet mental health needs and referring to a psychologist. According to the findings, a higher level of education in fathers (OR: 0.66; 95% CI: 0.50–0.87) and mothers (OR: 0.71; 95% CI: 0.55–0.92) as well as a higher level of SES (OR: 0.62; 95% CI: 0.47–0.81) was statistically associated with no unmet need for care, and these variables acted as a protective factor against experiencing an unmet need for mental health care. Smoking (OR: 3.34; 95% CI: 1.37–3.97), alcohol consumption (OR: 2.34; 95% CI: 1.44–3.76), and hookah consumption (OR: 1.69; 95% CI: 1.02–2.82) were statistically associated with the perception of the unmet need for mental health care. As shown in Table 3, there was no statically significant relationship between demographic and behavioral variables with referring to a psychologist.

**Table 3:** Factors associated with perceived unmet need for care and referred to a psychologist in all participants.

	Feeling the need for care			Referred to a psychologist		
	OR	95% CI	*p* value	OR	95% CI	*p* value
**Age**	1.07	0.94–1.21	0.285	1.09	0.96–1.25	0.166
**Sex**						
Female	Ref	–	–	Ref	–	–
Male	0.16	0.88–2.07	0.165	1.56	0.99–2.45	0.052
**Father’s education**	0.66	0.50–0.87	0.003^*^	1.20	0.90-1.60	0.205
**Mother’s education**	0.71	0.55–0.92	0.011^*^	1.16	0.88-1.53	0.267
**Socio-economic status**	0.62	0.47–0.81	0.001^*^	0.99	0.76-1.30	0.990
**Smoking**	3.34	1.37–3.97	0.002^*^	1.31	0.76-2.24	0.317
**Hookah**	1.69	1.02–2.82	0.043^*^	1.06	0.62-1.81	0.808
**Alcohol**	2.34	1.44–3.76	0.001^*^	1.45	0.89-2.35	0.128
**Drug use**	2.44	0.99–5.99	0.051	0.90	0.35–2.28	0.831
**Having a friend of the opposite sex**	1.39	0.90–2.15	0.138	1.32	0.82–2.05	0.245

*Significant at 0.05 level; CI: confidence interval.

**Figure 2:**
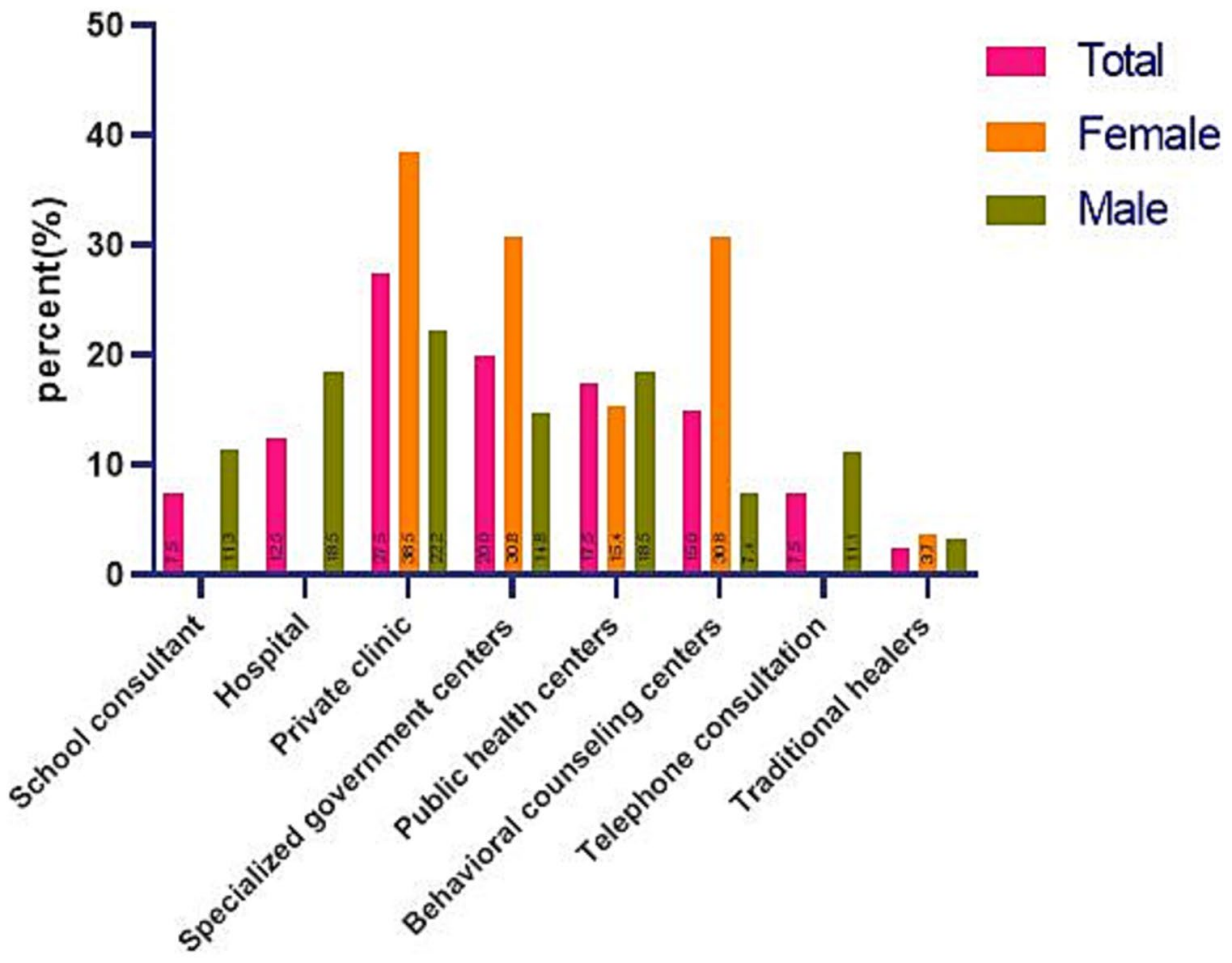
Referral places in participants who had referred to receive service.

Figure 2 illustrates the locations of the services received by the participants who were referred to a psychologist. The female and male adolescents were both more likely to seek care at private clinics. There was no statistically significant difference in the referral location between male and female adolescents.

**Figure 3:**
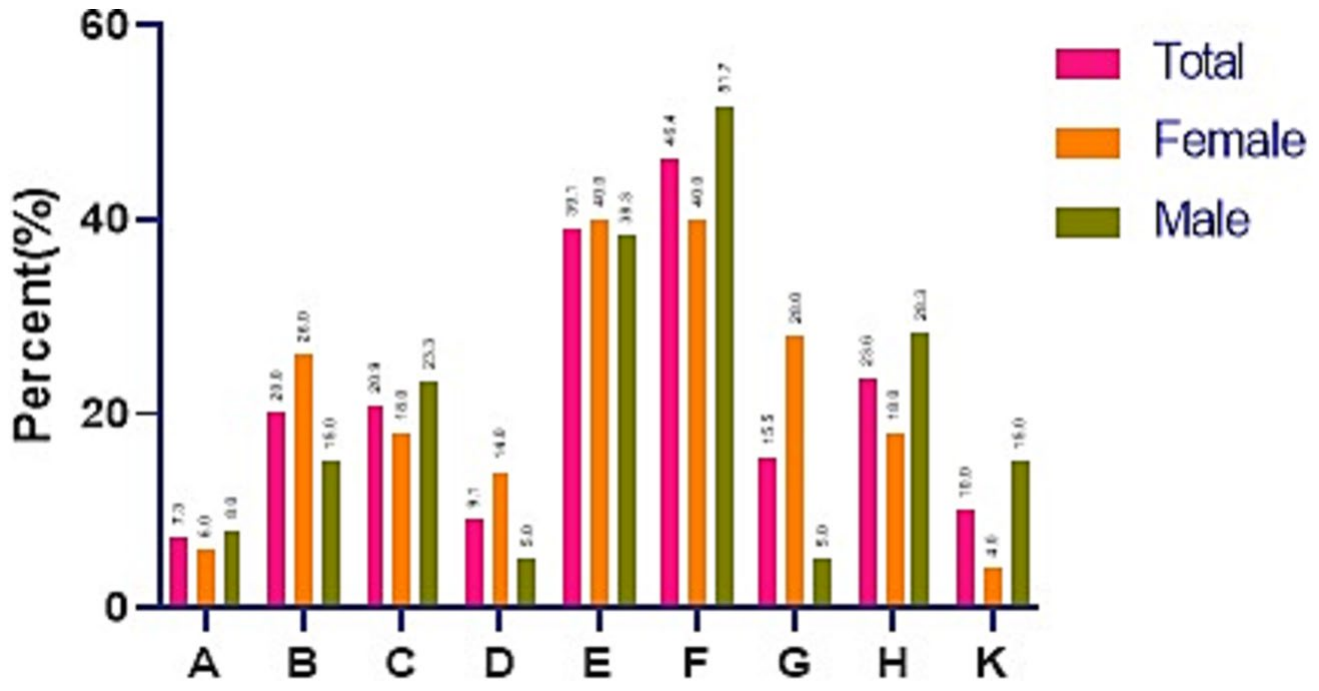
Structural and attitudinal barriers for receiving needed services in participants who perceive an unmet need. A: problem getting to a service that could help (transportation); B: lack of financial means or being unable to afford the money; C: low perceived efficacy of treatments; D: lack of insurance or insufficient coverage for services; E: asked but did not receive help; F: Reluctance to seek mental health care; G. not knowing where to get help; H: the presence of stigma (being afraid to ask for help); K: getting help from another source.

Figure 3 demonstrates, in details, the structural and attitudinal barriers for receiving needed services in participants who perceived an unmet need. In general, and in both male and female adolescents, *reluctance to seek mental health care* was a common barrier to care. The second barrier to service use was “asked but not receiving help.” The female adolescents were significantly more likely than the male adolescents to be unaware of where to get help (*p* = 0.001). Also, adolescents with lower SES were significantly unable to afford services (*p* = 0.002).

## Discussion

This study aimed to investigate unmet mental health needs among the adolescents living in Shiraz, Iran. According to the findings, 150 (43.1%) adolescents reported unmet mental health needs and 118 (33.9%) adolescents were referred to a psychologist and received some services. In total, 75 (21.6%) adolescents were classified as having wholly unmet needs, and 75 (21.6%) adolescents as having partially met needs. Although there are few recent studies to assess the prevalence of unmet mental health needs among adolescents, a study conducted in Australia revealed that 25.9% of adolescents were classified as having “wholly unmet needs” and 12.8% of adolescents were classified as having “partially met needs” (23). Also, Schnyder et al. showed that 9.1% and 6.9% of adolescents had “wholly unmet needs” and “partially met needs”, respectively (24).

The present study demonstrates that the mean score of all Schemas was significantly higher in adolescents with any unmet mental health need than those with no unmet mental health need. EMS can be explained as a dysfunctional cognitive and emotional pattern formed during childhood, which is repeated throughout an individual’s life, resulting in psychological disorders (4, 25). Likewise, the results of a systematic review in 2020 demonstrated a link between EMS and psychological pathology in adolescents (26). Numerous studies have shown a correlation between EMS and anxiety, stress, depression, and emotional disorders (27–30). It is evident that as mental disorders become increasingly prevalent, there arises a greater demand for individuals to seek mental healthcare services. The present study also revealed a significant positive association between some schemas and seeking mental health care; which means, as the score of certain schemas increases, there is a higher inclination towards seeking mental healthcare services. As evidence confirm, EMS are accompanied by a different range of psychopathological symptoms during lifetime that could be addressed required mental healthcare services (26). However, seeking mental health care could be affected by sociocultural context, accessibility, and affordability in addition to disease-related factors (3, 31). For instance, Muslim American immigrant parents and their children might have different cultural perspectives due to different adaptation level with the U.S. mainstream culture (32). This variation can play a significant role in seeking mental healthcare behavior of parents for their children. This variation can also be seen in Iran with a huge ethno-cultural and social diversity that, as the present study showed, could affect help-seeking behavior of mental healthcare.

Based on the present study, “Reluctance to seek mental health care,” “asking but not receiving help,” and “stigma” were the most significant attitudinal barriers to use services. The obstacles mentioned in the study sample can be attributed to the family’s cultural background and the adolescent’s social environment. Stigma, the fear of being labeled as mentally ill, the negative beliefs of those in their immediate environment, such as peers and families, as well as the fear of being negatively judged, prevented them from seeking treatment and follow-up. A qualitative study conducted among adolescents in Pakistan identified “awareness of mental health services and treatment options” and “stigma” as the main barriers to using MHS (13). Furthermore, negative past experiences in service utilization and help-seeking have been reported as an effective factor in their attitudes toward applying or using the services. Occasionally, the belief that “nothing and nobody can assist” encourages exploration, self-medication, and online search in the virtual world without the assistance of experts (33, 34). Studies conducted in Iran have estimated that 16.7 to 36.6% of children and adolescents suffer from one or more mental health problems, while there are very limited resources to meet this need. Despite a well-developed health network system for delivering primary care services in Iran, mental health care has remained irresponsive, especially in urban areas (35). As pieces of evidence show, integrating adolescent-oriented mental health care to prevent and manage mental health problems among these age groups into primary care services would be the best strategy to address the need (31).

In line with other studies, other attitudinal factors reported by the adolescents, such as low efficacy of mental health care and not knowing where to get help, are rooted in their insufficient information, knowledge, and undefined adolescent-oriented MHS (36).

From a structural point of view, socioeconomic factors such as “insufficient insurance coverage for MHS,” and “lack of financial means or unaffordability” were important barriers for seeking care. The present study revealed that adolescents with lower SES did not apply for MHS due to the high cost of care. It is worth noting that most of mental care costs are not covered in Iran (37). Similarly, Lubman et al. (38) and Hernan et al. (39) found that economic barriers are a significant reason why people avoid MHS in Australia. Furthermore, a study conducted by Cullinan et al. (40) on 6,000 high school students in Ireland found that socioeconomic inequalities play an essential role in the unmet needs of adolescents, although adolescents with the lowest S.E.S have a higher rate of unmet needs.

According to the present study, adolescents prefer private clinics over government-run or behavioral counseling centers. The female adolescents were more likely to visit private clinics than the male adolescents. It is implied from our findings that adolescents, particularly females, may view private clinics as secure and safe environments where mutual respect and confidentiality are emphasized. As a result, they had a strong disinclination to visit other centers. Establishing a trusting relationship between adolescents, counselors, and staff is critical in encouraging adolescents to visit a mental health center that offers services. Based on previous research, adolescents avoid seeking counseling due to concerns about the privacy and confidentiality of their problem and its management (41, 42).

In this study, adolescents who used alcohol, cigarettes, and psychotropic drugs significantly had unmet needs for MHS as compared with those who had not. Consumption of alcohol, tobacco, and psychotropic drugs may be considered as risk factors for adolescents, and show that they need counseling and MHS. In the United States, an online survey of 2,843 students revealed that 67% of those who consume alcohol or drugs recognize the need for MHS, but only 38% seek treatment (43). Also, adolescents whose parents had a higher level of education significantly reported no unmet needs for MHS. A systematic review found that parents’ perceptions of their children’s need for MHS, family formation (including single-parent or two-parent families), and their level of mental health-related education all affect an adolescents’ need for MHS. Moreover, parents’ level of education and income could be influential (44). It also showed that parental education helped parents’ recognition concerning their children’s need to MHS. Nonetheless, parents’ income and level of education did not always predict their adolescent’s level of mental health education and perception of the need for services (44).

To the best of the authors’ knowledge, this is the first study conducted in Iran with a focus on unmet mental health needs in adolescents and related contributing factors to seek MHS. Therefore, it would be advisable to run similar study in other parts of the country to find a national map of this issue for accurate response. Also, since this work coincided with the COVID-19 pandemic, it was challenging to access adolescents through schools, so we use convenience sampling method that would be a weakness for this study. To address this problem, various locations were visited throughout Shiraz, encouraging adolescents to cooperate and earning their trust by assuring the confidentiality of their information.

## Conclusion

The present study shows that approximately half of the adolescents have unmet mental health needs, but less than a quarter actually use some services. The most significant impediments preventing adolescents from using the relevant services were identified as attitudinal as well as structural obstacles. The results of this study provide health policymakers with valuable evidence to focus on addressing the needs of an important target population. This requires strengthening primary care settings to establish adolescent-oriented mental health programs, and promote the mental health literacy of families and adolescents to reduce the stigma attached to mental disorders and improve the use of these services. To do this, several strategies would be advisable including using the capacity of well-established national health network system with a focus on its skilful community health workers, mobilizing school-based initiatives such as parents-teachers association, and using community outreach through voluntary programs. Health policymakers should improve accountability in the health system to ensure the comprehensiveness and effectiveness of these services to adolescents with the focus on primary health care. They should also take some measures concerning economic barriers, such as providing insurance coverage, lowering expenses, and reducing economic inequalities.

## Data availability statement

The raw data supporting the conclusions of this article will be made available by the authors, without undue reservation.

## Ethics statement

The studies involving humans were approved by Ethics Committee of Shiraz University of Medical Sciences. The studies were conducted in accordance with the local legislation and institutional requirements. Written informed consent for participation in this study was provided by the participants’ legal guardians/next of kin.

## Author contributions

HJ: Conceptualization, Methodology, Supervision, Writing – review & editing. MF: Conceptualization, Methodology, Data curation, Writing – original draft. RJ: Data curation, Methodology, Writing – original draft. MH: Methodology, Writing – review & editing. AP: Writing – review & editing, Data curation. ZF: Conceptualization, Formal analysis, Methodology, Writing – original draft.
